# Antibacterial Films Based on Polylactide with the Addition of Quercetin and Poly(Ethylene Glycol)

**DOI:** 10.3390/ma14071643

**Published:** 2021-03-27

**Authors:** Ewa Olewnik-Kruszkowska, Magdalena Gierszewska, Agnieszka Richert, Sylwia Grabska-Zielińska, Anna Rudawska, Mohamed Bouaziz

**Affiliations:** 1 Faculty of Chemistry, Chair of Physical Chemistry and Physicochemistry of Polymers, Nicolaus Copernicus University in Toruń, Gagarin 7 Street, 87-100 Toruń, Poland; mgd@umk.pl (M.G.); sylwia.gz@umk.pl (S.G.-Z.); 2 Faculty of Biological and Veterinary Sciences, Chair of Genetics, Nicolaus Copernicus University in Toruń, Lwowska 1 Street, 87-100 Toruń, Poland; a.richert@umk.pl; 3 Faculty of Mechanical Engineering, Department of Production Engineering, Lublin University of Technology, 20-618 Lublin, Poland; a.rudawska@pollub.pl; 4 Electrochemistry and Environmental Laboratory, National Engineering School of Sfax, University of Sfax, BP1173, Sfax 3038, Tunisia; mohamed.bouaziz@fsg.rnu.tn

**Keywords:** polylactide, quercetin, antibacterial properties, food packaging

## Abstract

A series of new films with antibacterial properties has been obtained by means of solvent casting method. Biodegradable materials including polylactide (PLA), quercetin (Q) acting as an antibacterial compound and polyethylene glycol (PEG) acting as a plasticizer have been used in the process. The effect of quercetin as well as the amount of PEG on the structural, thermal, mechanical and antibacterial properties of the obtained materials has been determined. It was found that an addition of quercetin significantly influences thermal stability. It should be stressed that samples containing the studied flavonoid are characterized by a higher Young modulus and elongation at break than materials consisting only of PLA and PEG. Moreover, the introduction of 1% of quercetin grants antibacterial properties to the new materials. Recorded results showed that the amount of plasticizer did not influence the antibacterial properties; it does, however, cause changes in physicochemical properties of the obtained materials. These results prove that quercetin could be used as an antibacterial compound and simultaneously improve mechanical and thermal properties of polylactide-based films.

## 1. Introduction

In recent years, polymers have become one of the most common materials used for packaging, replacing materials such as glass, metal, paper and wood. It is estimated that about 40% of industrial packaging and about 50% of consumables packaging is made of polymers. Such a large interest in plastics as packaging materials is due to their many beneficial properties, such as low permeability and high mechanical strength [1,2,3].

Packaging materials, in particular those intended for food, have to meet a significant number of requirements. Most of all they have to ensure effective protection of the product against harmful factors (such as oxygen, UV radiation, moisture, bacteria and fungi) during food transport, storage and shelf life. For this reason materials with an additional function of so called active packaging are becoming more popular. The most popular include polymers modified with various substances characterized by such properties as absorption of moisture, oxygen or carbon dioxide. The group of active agents being introduced into the polymer matrix also includes substances with biocidal properties. The main benefit of using food packaging materials with antibacterial properties is the extension of products’ shelf life and protection against bacteria and fungi, which can be hazardous to human health [2,4,5,6].

Currently, materials utilized in the production of packaging consist mainly of polyethylene, poly(ethylene terephthalate), polypropylene, poly(vinyl chloride) and polystyrene. Unfortunately, the use of polymer materials leads to the accumulation of huge amounts of waste that are harmful to the natural environment. This is due to their high durability and inherent resistance to biodegradation. Plastic waste accounts for about 30% of the weight of all the waste in the world. The growing problem of waste has recently resulted in an increased popularity and interest in biodegradable polymers. It seems to be very likely that in the immediate future they may completely replace traditional polymer materials. One of the polymers that will in all probability become an alternative to the currently used polymers of petrochemical origin is polylactide (PLA) [7,8]. It has to be stressed that polylactide is an aliphatic biodegradable polyester that can be obtained from renewable raw materials of plant origin [9]. The most popular materials used in an aim to manufacture lactic acid include corn, rice, barley, wheat or cassava [10].

In order to obtain antibacterial packaging materials based on polylactide, different compounds such as nanosilver, nisin, polyhexamethylene guanidine derivatives, cinnamon or tea tree essential oil [11,12,13,14,15,16] have been used. Among different active substances, flavonoids constitute a group of very interesting compounds characterized by varied antibacterial properties.

Molecular structure of flavonoids is characterized by two aromatic rings, which are connected by a three-carbon bridge. In the group of flavonoids we can distinguish: flavones, isoflavones, flavonols, flavanones, flavanols and anthocyanins. Flavonoids can, for example, be found in leaves, flowers, plant seeds, fruit (e.g., in citrus fruits, blueberries, berries, grapes, chokeberry), vegetables (e.g., in peppers, onions, tomatoes, broccoli) and in coffee and cocoa beans. It has to be noted that they possess many beneficial properties. They are characterized by anti-inflammatory, antiviral, antiatherosclerosis, antiallergic and anti-cancer properties. In addition, they perform a protective function, for instance deterring insects and inhibiting fungi, a common hazard to plants in which flavonoids can be found. The presence of these compounds also hinders the adverse effects of ultraviolet radiation [17,18,19].

**Figure 1:**
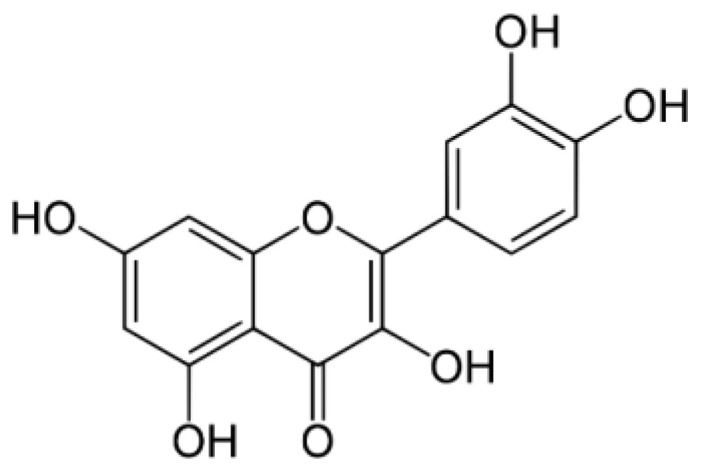
The structure of quercetin.

One of the most popular flavonoids is quercetin—3,3′,4′,5,7-pentahydroxylflavone (Figure 1). It can be found in many types of fruit, vegetables, leaves, seeds and grains. Quercetin is also present in medicinal botanicals, including Ginkgo biloba and in varieties of honey from different plant sources. Recently scientists focused on its biological and antioxidative characteristics [20,21,22,23]. Moreover, it should also be mentioned that the flavonoid in question was used in the formulation of packaging films consisting mainly of ethylene-vinyl alcohol copolymer (EVOH) [24]. It was also scrutinized as an antioxidant and environmentally-friendly colored indicator of ageing time in a topas cyclo-olefin copolymer (ethylene-norbornene) [25]. Additional research devoted to the possible applications of quercetin has been described in the work of Kost et al. [26], where the fibers based on polylactide and β-cyclodextrin loaded with quercetin were used as dressing materials characterized with antibacterial properties. Other possible applications of the quercetin have been presented in the work of Hao et al. [27], where quercetin was encapsulated using chitosan-coated nanoliposomes.

In the present work polylactide-based films with an addition of poly(ethylene glycol) acting as a plasticizer and quercetin used as a biocidal agent are suggested as new antibacterial packaging materials. Taking into account the properties of quercetin-infused PLA films, it has to be stressed that they present an enormous potential for application in the food industry.

## 2. Materials and Methods

### 2.1. Materials

Polylactide, type 2002D with melt flow rate 5–7 g/10 min (2.16 kg; 190 °C), with average molecular weight of 155,500 Da and content of monomeric units D and L equal to 3.5% and 96.5%, respectively was delivered in the form of pellets by Nature Works^®^ (Minnetonka, MN, USA). Quercetin, an antibacterial compound, was purchased from Sigma-Aldrich (Steinheim, Germany). Poly(ethylene glycol) with an average molecular weight of 1500 (Sigma-Aldrich, Steinheim, Germany) was applied as the plasticizing agent. Acetone and chloroform were purchased from Avantor Performance Materials Poland S.A. (Gliwice, Poland).

In an aim to analyze antibacterial properties of the obtained materials, two bacterial reference strains were used in the study: Escherichia coli (ATCC 8739) and Staphylococcus aureus (ATCC 6538P) (Microbiologics^®^, St. Cloud, MN, USA). Moreover, an agar medium containing a composition [g/L]: tryptone peptone—15, phyton peptone—5, sodium chloride—5, agar—agar—15 was acquired from Oxoid (Hampshire, UK).

### 2.2. Formation of PLA-Based Films

In aim to obtain all of the PLA-based films, the solvent evaporation technique were applied. In the first stage, 1.5 g of pure and dry polylactide was dissolved in 35 mL of chloroform by vigorous mixing at room temperature. In the second stage, an appropriate amount of PEG (5% or 10% *w*/*w* of PLA, 0.075 g or 0.15 g of PEG, respectively) was added. Quercetin was dissolved in 15 mL of acetone and the mixture was then introduced into the solution containing PLA and PEG. The resulting solutions were cast onto clean glass plates, 145 mm in diameter and dried at ambient temperature for 48 h. Designations and compositions of the obtained materials are presented in Table 1.

### 2.3. Fourier Transform Infrared Analysis Analysis

The Fourier transform infrared analysis of all studied materials was performed by means of a Nicolet iS10 (Thermo Fisher Scientific, Waltham, MA, USA). The spectra were recorded in the frequency range of 500–4000 cm^−1^ at a resolution of 4 cm^−1^ and scanned 64 times. The spectrum of quercetin in KBr disc form was obtained in the same conditions. All spectra were analyzed using the OMNIC 7.0 software (Thermo Fisher Scientific, Waltham, MA, USA).

### 2.4. Scanning Electron Microscopy (SEM)

Changes in the structure of the PLA-based films caused by an addition of PEG and quercetin were analyzed using a scanning electron microscope (Quanta 3D FEG, FEI Company, Hillsboro, OR, USA). In an aim to obtain high quality images, the samples were covered with a thin layer of gold. Images of all samples were taken at 5000× magnification.

### 2.5. Atomic Force Microscopy (AFM)

The surface pictures of the PLA-based films were obtained by means of an AFM microscope with a scanning probe of the NanoScope MultiMode type (Veeco Metrology, Inc., Santa Barbara, CA, USA). Analyses were performed in the tapping mode, in air, at room temperature. Using a scan area of 5 × 5 µm and Nanoscope software (Veeco Metrology, Inc. Santa Barbara, CA, USA), the roughness parameters such as the root mean square (*R_q_*) and arithmetical mean deviation of the assessed profile (*R_a_*) were calculated.

### 2.6. Thermogravimetric Analysis

Thermogravimetric (TG) analyses of the PLA films with an addition of PEG and quercetin were performed on Simultaneous TGA-DTA Thermal Analysis type SDT 2960 (TA Instruments, London, UK). All measurements were carried out at a heating rate of 10 °C/min under air flow from room temperature to 600 °C.

### 2.7. Differential Scanning Calorimetry Method

Thermal analyses were carried out on DSC (Polymer Laboratories, Epsom, UK). Experiments were performed under nitrogen screening. The thermal response in the obtained materials was investigated at the temperatures ranging from 25 °C to 180 °C (1st heating cycle) and with a heating rate of 10 °C/min. The PL5 software version v5.40 (Polymer Laboratories, Epsom, UK) was applied in an aim to establish the detailed information extracted from the DSC data. The degree of crystallinity (*X_m_*) was established by applying the following Equation (1) [28,29]:

(1) $$X_{m}=\frac{∆H_{m}}{∆H^{0}\cdot X_{PLA}}\cdot 100\%$$

where Δ*H_m_* is the measured heat of fusion of sample, Δ*H*^0^ is the heat of fusion of a 100% crystalline polylactide and Δ*H*^0^ = 109 mJ/mg, *X_PLA_* is the mass fraction of polylactide.

### 2.8. Mechanical Properties

The mechanical properties of the PLA-based films with and without an addition of quercetin were analyzed by means of the Instron 1193 machine (Instron Corp., Canton, OH, USA) test according to the PN-EN ISO 527-1, -3 standard [30,31]. The crosshead speed was 20 mm/min with an applied 100 N force. In the case of each type of the studied materials, at least five samples were analyzed. The obtained results allowed to establish the Young’s modulus (*E*), elongation at break (*ε*) and tensile stress (*σ_m_*).

### 2.9. Transparency

Transparency of the obtained materials was established based on the method presented in work of [32]. Absorbance of the polymeric films with and without quercetin at 600 nm ($A_{600}$) was measured by means of UV spectrophotometer (Ruili Analytical Instrument Company, Beijing, China). The analysed films were placed directly in a spectrophotometer test cell while an empty cell was used as the blank. The transparency (*T*) of the obtained materials was calculated according to the following Equation (2):

(2) $$T=\frac{A_{600}}{d}\;\;\left[mm^{-1}\right],$$

where *d* is the film thickness [mm]. It should be noted that a higher transparency value is equivalent to lower transparency.

### 2.10. Colour Measurement

The changes in the color of the obtained films caused by the introduction of quercetin and PEG were studied by means of a MICRO-COLOR II LCM 6 (Dr Lange, Berlin, Germany) colorimeter. The CIE L*a*b* system was applied in aim to calculate the colour difference (Δ*E*) of materials. The following Equations (3) and (4) were used in order to establish the total values of Δ*E* and color intensity (*C*), respectively.

(3) $$∆E=\sqrt{{\left(L-L^{*}\right)}^{2}+{\left(a-a^{*}\right)}^{2}+{\left(b-b^{*}\right)}^{2}},$$

(4) $$C=\sqrt{{\left(a^{*}\right)}^{2}+{\left(b^{*}\right)}^{2}},$$

where *L* is the component describing lightness, a represents the colors ranging from green (*−a*) to red (*+a*), while *b* represents a parameter change from blue (*−b*). The color of the control film (in this case a LP5 film), was expressed as L* (lightness), $a^{*}$ (redness/greenness) and $b^{*}$ (yellowness/blueness) values [14]. In an attempt to obtain reliable data at least five measurements were performed for each of the samples, then average values were calculated. Moreover, based on the obtained results yellowness index (*YI*) was established using the Equation (5) described in the work of Pathare et al. [33]:

(5) $$YI=\frac{142.86\cdot b^{*}}{L^{*}}.$$

### 2.11. Analysis of Antibacterial Properties

Antibacterial properties were determined according to the ISO 20645:2006 standard: “Flat textile products. Determination of antibacterial activity. Diffusion method on an agar plate” [34]. Agar medium consisting of [g/L]: tryptone peptone—15, phyton peptone—5, sodium chloride—5, agar—agar—15 was poured onto each petri dish and allowed to gel. The medium was then infused with a bacterial culture at a concentration of 1.5 × 10^8^ cfu/mL (0.5 McFarlanda). Centrally tested samples and control samples in the shape of a circle with a diameter of 25 ± 5 mm (four replicates) were placed on the dishes prepared in this way. Plates were incubated for 20 h at 37 ± 1 °C. After the end of the incubation time, the presence or absence of zones inhibiting the growth of microorganisms was determined. The width of the braking zone (*H*), i.e., the zone without bacteria near the edge of the sample, was calculated using the following Formula (6):

(6) $$H=D-\frac{d}{2}\;\;\left[\mathrm{mm}\right],$$

where:

*H*—braking zone width [mm],

*D*—total diameter of the working sample and width of the braking zone [mm],

*d*—diameter of the working sample [mm].

Stereoscopic microscope SZX 12 (Olympus, Tokyo, Japan) was used in an aim to establish the size of inhibition zones of bacterial growth as well as the extent of microorganism proliferation in contact of the studied films with agar. The appearance of agar plates after the studied materials had been removed was recorded by means of a SCAN^®^ 1200 colony counter (Interscience, Saint-Nom-la-Bretèche, France). The scale shown in Table 2 was used to assess the potency of antibacterial properties.

## 3. Results and Discussion

### 3.1. FTIR Analysis

**Figure 2:**
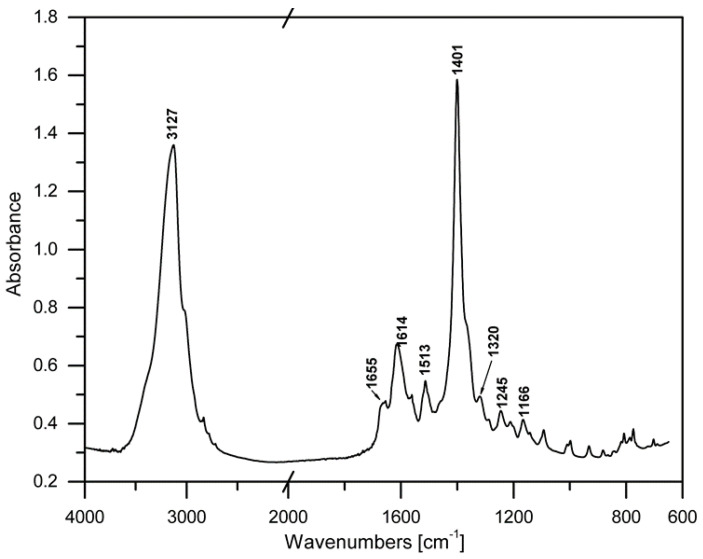
The FTIR spectrum of quercetin.

**Figure 3:**
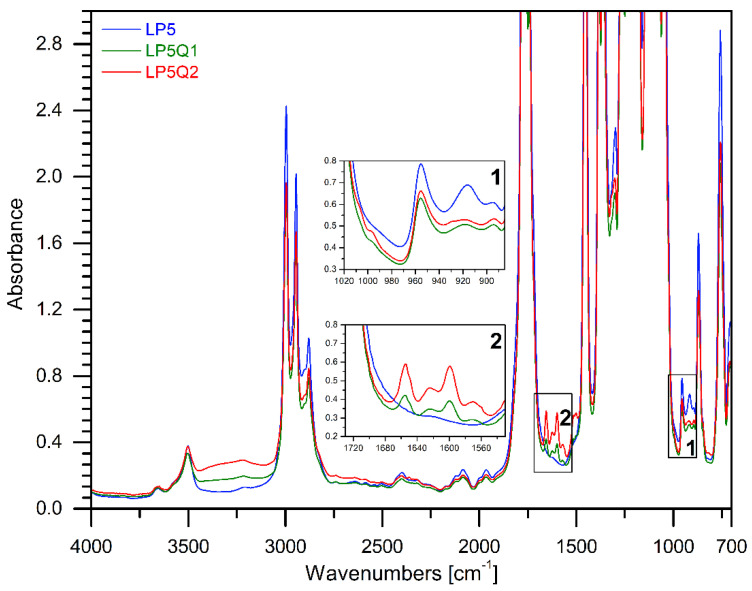
The FTIR spectra of materials consisting of PLA, PEG 5 wt.% with an addition of quercetin.

**Figure 4:**
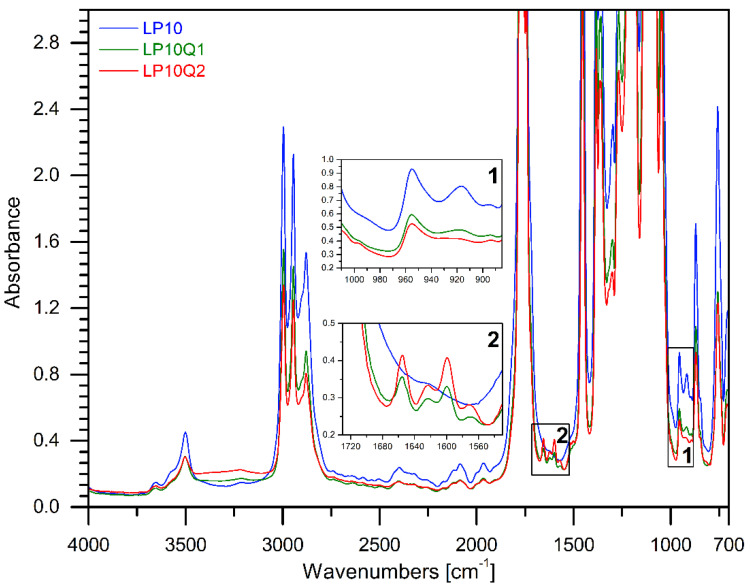
The FTIR spectra of materials consisting of PLA, PEG 10 wt.% with an addition of quercetin.

FTIR spectroscopy was used in an aim to establish the structure of the obtained materials with an addition of quercetin and different amounts of PEG. The FTIR spectrum of quercetin is shown in Figure 2, where the bands of characteristic groups can be observed. According to the literature [35,36] particular bands were assigned to the characteristic groups present in the quercetin structure. A broad band with the maximum at 3127 cm^−1^ belongs to the hydroxyl groups (phenolic -O-H stretching). The band at 1653 cm^−1^ indicates the presence of stretching vibrations of the -C=O group, while the bands at 1614 cm^−1^, 1567 cm^−1^ and 1513 cm^−1^ belong to the C=C stretching bonds in the aromatic rings of quercetin [36,37]. In the spectrum of the neat quercetin an intense band at 1401 cm^−1^ is visible and can be ascribed to deformation vibrations of -OH groups. Additionally, in the discussed spectral bands, the maximum values of 1316 cm^−1^ and 1247 cm^−1^ correspond to the deformation vibrations of the -C-OH group. Other vibrations at 1167 cm^−1^ and 1092 cm^−1^ can be assigned to the anti-symmetrical and symmetrical stretching vibration of the -C-O-C group. The band observed at 997 cm^−1^ indicates the deformation vibrations of the -OH group, while the band at 933 cm^−1^ relates to the stretching vibrations of the -C-O. Other characteristic bands in the range between 884 cm^−1^ and 808 cm^−1^ correspond to the deformative vibrations of the -CH groups [35,37]. In Figure 3 and Figure 4, the spectra of the materials consisting of PLA, PEG with or without addition of quercetin are depicted. The chemical structure of polylactide films is well known. It was also described in our previous publications [38]. Based on our study, it can be clearly seen that the bands at 3657 cm^−1^ and 3502 cm^−1^ correspond to -OH groups at the end of PLA chains. Absorption bands at 2996 cm^−1^ and 2947 cm^−1^ belong to the symmetrical and asymmetrical stretching vibrations of the -CH_3_ group, while the band at 1763 cm^−1^ relates to the characteristic -C=O carbonyl group. Moreover, symmetrical stretching vibrations of -C-O-C were recorded at 1207 cm^−1^ and at 1127 cm^−1^. The bands describing the stretching vibration of C-COO and the deformation vibration of CO can be seen at 873 cm^−1^ and 756 cm^−1^.

In the case of films based on PLA and PEG the typical vibrations caused by the -CH_2_ group at 2878 cm^−1^ can be observed. Moreover, a peak at 3446 cm^−1^ for poly(ethylene glycol), which corresponds to the terminal hydroxyl group, has been recognized. Furthermore, it can be clearly noticed that the intensity of the band at 2878 cm^−1^ increases with the increase in the amount of PEG.

Introduction of quercetin into the PLA-PEG systems reveals a broad band at 3300 cm^−1^ which can be assigned to the -OH groups present in the structure of quercetin. Moreover, new bans at 1655 cm^−1^, 1615 cm^−1^ and 1600 cm^−1^ corresponding respectively to stretching vibrations of the -C=O group and the C=C stretching bonds in the aromatic rings have been recognized. It has to be stressed that the intensities of the described new bands increased with the quantity of quercetin in the obtained films [37].

The spectra of the studied films after the addition of quercetin present certain changes in the intensity of bands at 915 cm^−1^ and 955 cm^−1^ belong to the amorphous and crystalline phases of PLA, respectively [39,40]. Increase in intensity of band at 915 cm^−1^ indicates that quercetin influences the crystallinity of the obtained materials.

### 3.2. SEM and AFM Analyses

**Figure 5:**
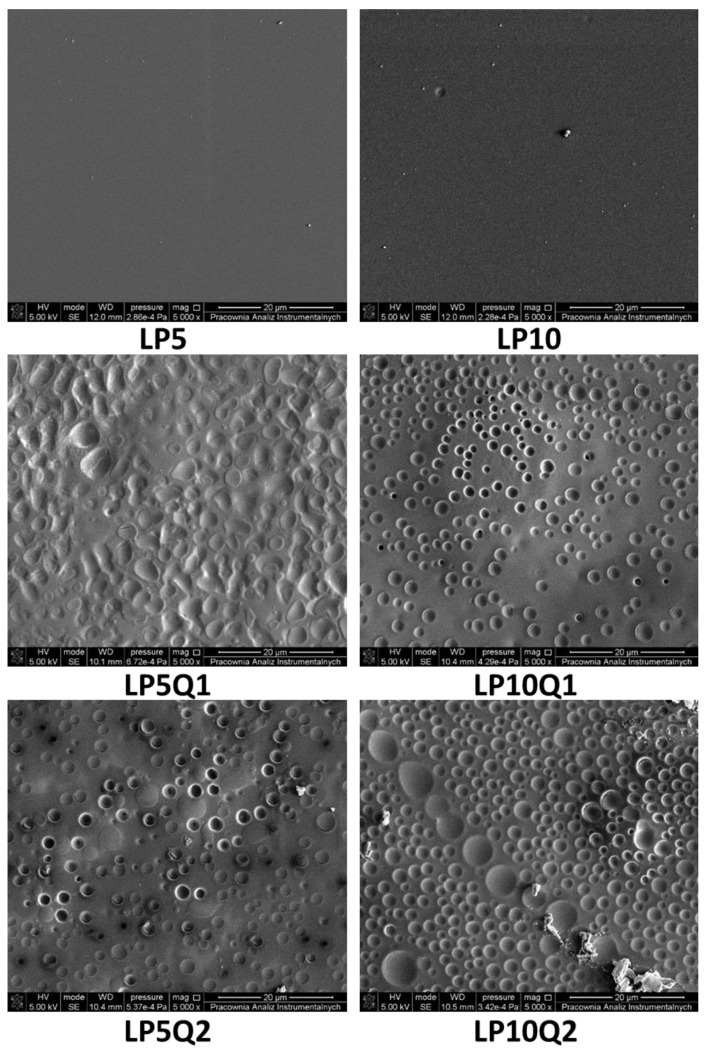
SEM images of obtained materials.

Topography and surface morphology constitute factors which are taken into account when the potential application of materials is discussed. They are extremely important in the case of polymeric films used as food packaging as well as films with medical applications. In the present work we focused on the PLA-based materials with an addition of quercetin and poly(ethylene glycol). Obtained materials were characterized by antibacterial properties. In order to analyze their surface morphology atomic force microscopy and scanning electron microscopy were applied. The SEM images presented in Figure 5 reveal that samples of polylactide with an addition of 5 wt.% or 10 wt.% of poly(ethylene glycol), which plays the role of a plasticizer, were characterized by a smooth and flat surface, without any cracks or fissures. The same observation was made in the work of Jasim Ahmed at al. [41] where PLA-based films with an addition of 20 wt.% of PEG were studied. In the mentioned work, SEM photographs depicted good dispersion of PEG into the polylactide matrix, indicating that the obtained materials form homogeneous blends. Incorporation of quercetin into the PLA-PEG blends significantly influences the morphology of the obtained polymeric films It has to be taken into account, however, that the surfaces of films with a higher amount of PEG (10 wt.%) are characterized by larger convexities and deeper concavities of various shapes, evenly distributed throughout the surface in comparison with materials containing 5 wt.% of plasticizer.

**Figure 6:**
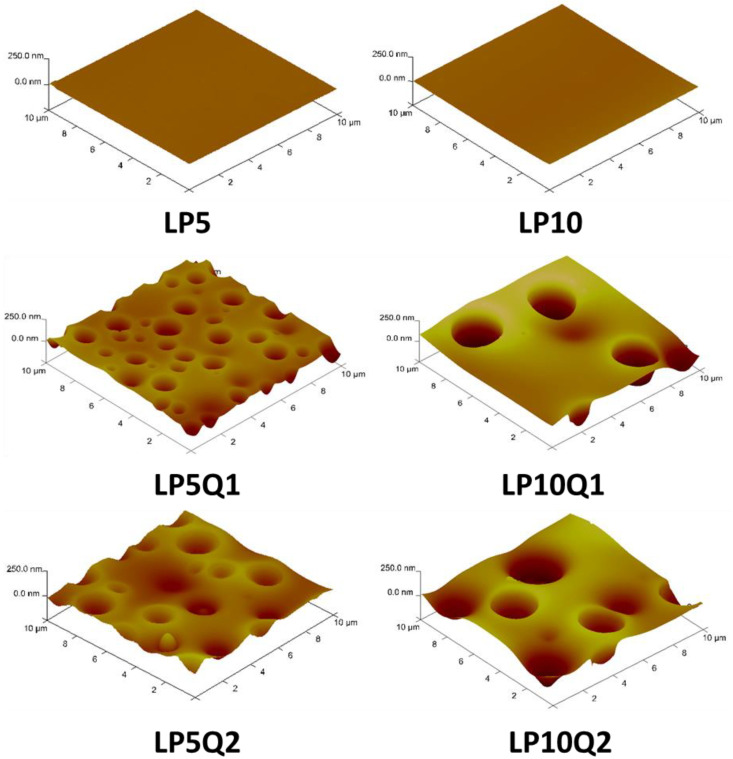
3D AFM images of obtained materials.

In an aim to establish the size and roughness of the studied surface, the AFM technique was applied. It is well known that roughness constitutes one of the factors which can significantly affect the antibacterial properties of the obtained materials. In Figure 6, three dimensional pictures of surfaces of PLA-based films are presented. It can be observed that the size of cavities formed on the surface of the obtained polymeric films containing quercetin, with an addition of 10 wt.% of PEG, are significantly enlarged. The diameter of the cavities in the case of LP5Q1 and LP5Q2 samples equaled 1.9 µm and 2.0 µm, respectively, while in the case of LP10Q1 and LP10Q2 materials the fissures measured 2.5 µm and 2.8 µm. Based on the presented images (Figure 6), the minimum depth of the formed cavities was also calculated. In the case of materials containing 5 wt.% of PEG (LP5Q1 and LP5Q2) the values of cavities’ depth equaled 57 nm and 75 nm, while in the case of films with an addition of 10 wt.% of the plasticizer (LP10Q1 and LP10Q2) the depth of cavities increased to an extent of 60 nm and 80 nm, respectively. The results presented above confirm that the diameter of the formed fissures increases with the increase in the amount of PEG, while the depth of observed cavities increases with the increase in the amount of quercetin.

Based on the obtained results the roughness parameters such as *R_q_* (mean square deviation of surface roughness), *R_a_* values (mean arithmetic deviation of the profile from the mean line) and *R_max_* (maximum distance between the highest and lowest point of the recorded image) have been discussed (Table 3) [16].

It needs to be stressed that the values of all of the analyzed roughness parameters significantly depend on the amount of PEG. Obtained results suggest a dependence between the increase of the amount of PEG and the roughness parameters discussed above which are characterized by significantly higher values. Taking into account the amount of quercetin used, values of *R_a_*, *R_q_* and *R_max_* reveal that the same tendency as in the case of PEG. The introduction of 2 wt.% of quercetin, however, leads only to a slight increase in the values of roughness parameters in comparison with the change of values caused by the plasticizer.

### 3.3. Determination of Thermal Properties of Polylactide-Based Materials

**Figure 7:**
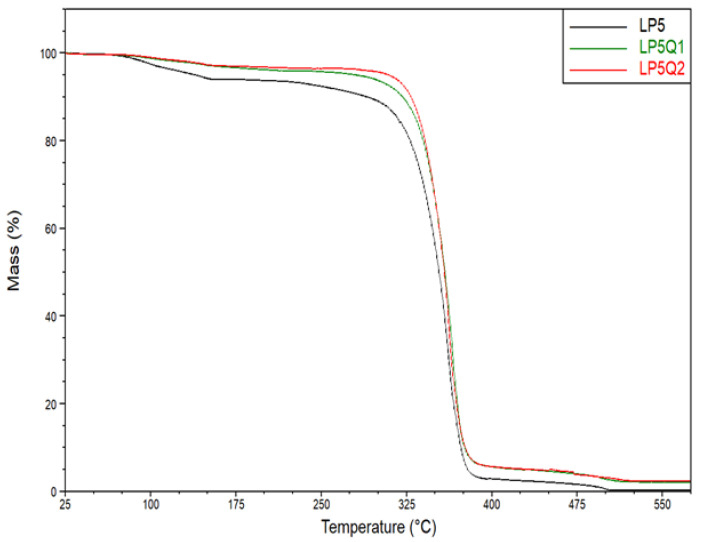
Thermogravimetric curves for polylactide-based materials with 5 wt.% of PEG.

**Figure 8:**
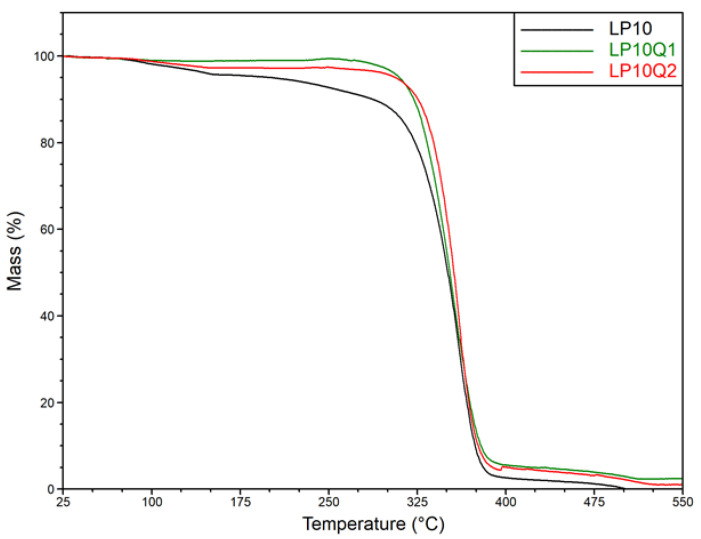
Thermogravimetric curves for polylactide-based materials with 10 wt.% of PEG.

In an aim to determine the effect of different amounts of plasticizer and quercetin on thermal stability of polylactide-based films, thermogravimetric analysis was conducted. Based on the obtained results, thermogravimetric (TG) curves for the samples LP5, LP5Q1, LP5Q2 are shown in Figure 7, while the TG curves relating to the samples LP10, LP10Q1 and LP10Q2 are depicted in Figure 8.

It was previously discussed that the plasticizer as well as the biocidal agent can significantly affect the thermal stability of the polylactide-based films [16]. In the prevailing number of publications the temperatures at 5% mass loss are analyzed. In the work of Rhim et al. [42] and Tarach at al. [16], however, it was indicated that in the case of these types of materials, the mass loss of about 5% of the initial mass is connected with the evaporation of the solvent residue. As a result, the values of temperatures at 10%, 30% and 50% mass loss of all studied materials (signified as T10%, T30% and T50%, respectively) were selected and presented in Table 4. Taking into account the amount of the plasticizer used, it can be observed that with an increase in the amount of PEG the thermal stability of the PLA-PEG samples decreases. According to the work of Phaechamud [43], as well as Pielichowski [44], the observed reduction in thermal stability is the result of PEG decomposition, which occurs at a significantly lower temperature compared to polylactide.

The incorporation of quercetin into the materials consisting of PLA and PEG, contributed to an increase in thermal stability of the obtained films. In thermograms presented in Figure 7 and Figure 8 relating to the materials with the addition of 5 wt.% of PEG and 10 wt.% of plasticizer, respectively, a significant increase in thermal stability with the increase in an amount of biocidal agent can be observed. In the case of samples LP5Q1 and LP5Q2, the temperature for a 10% mass loss, compared to the LP5 sample, increased by 33.1 °C and 40.3 °C, respectively. The same tendency was observed in the case of samples based on PLA with 10 wt.% of PEG and containing quercetin. The 10% loss of mass in films containing 10 wt.% of PEG and modified with 1 and 2 wt.% of quercetin occurred at the temperature 32.8 °C and 38.3 °C higher than in the case of the PL10 material. The obtained results indicate that the most significant changes in thermal stability of the obtained materials can be observed after the addition of 1 wt.% of quercetin. The abovementioned results reveal that after incorporation of 2 wt.% of biocidal agent into the PL5 as well as the PL10 systems, the values of T10%, T30% and T50% also increase. The changes between 1 wt.% and 2 wt.% of quercetin, however, are not as significant as after addition of 1 wt.% of quercetin. It should be mentioned that an improvement of thermal stability of the obtained materials after the addition of quercetin was also observed in the work of Samper et al. [45]. In the mentioned work an addition of 0.25 wt.% of quercetin resulted in a remarkably higher stability of polypropylene (PP) in comparison with non-stabilized PP. According to the literature [37,45] this phenomenon can be attributed to the structure of the phenolic compound used in the procedure, especially to the number of hydroxyl groups. Moreover, it has been proved that in the case of quercetin there is an optimum amount which provides the best thermal stability. Further introduction of this substance beyond the optimum point does not provide additional benefits in terms of structural stabilization. Summarizing, based on the analysis of the above mentioned data (Table 4, Figure 7 and Figure 8), it is reasonable to assume that the incorporation of quercetin significantly influences thermal stability of obtained materials. Obtained results are consistent with the data presented by the researchers mentioned above.

**Figure 9:**
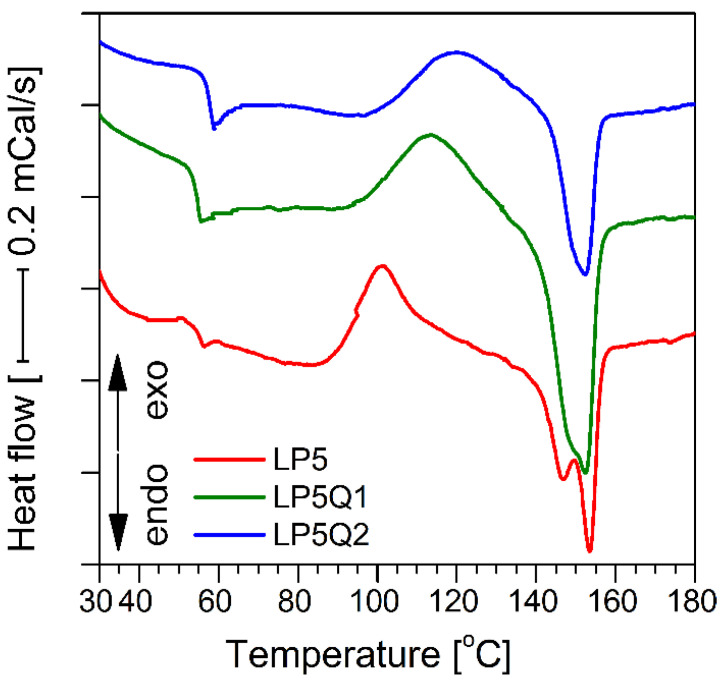
DSC thermograms of LP5 with and without an addition of quercetin.

**Figure 10:**
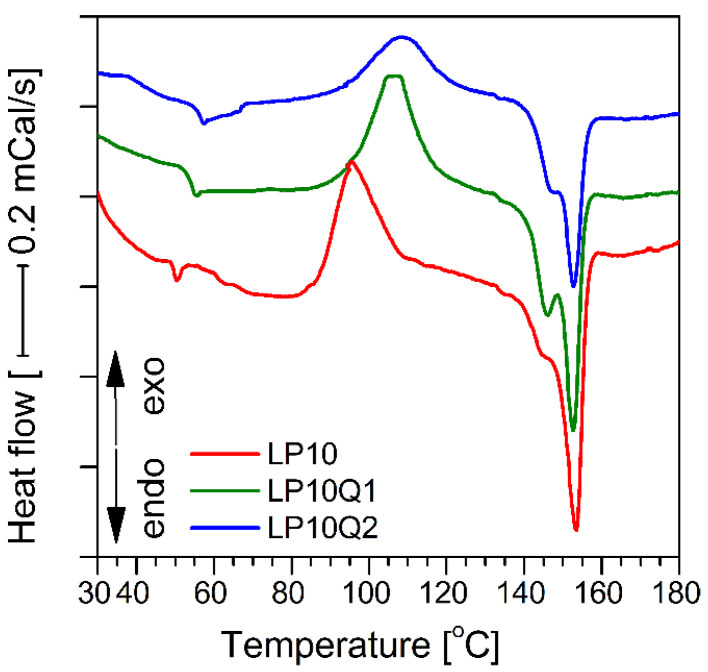
DSC thermograms of sample LP10 with and without an addition of quercetin.

In order to determine changes in thermal parameters of the studied materials, caused by the addition of a plasticizer and quercetin, differential scanning calorimetry has been carried out ( Figure 9 and Figure 10).

The obtained parameters of the glass transition temperature (*T_g_*), crystallization temperature (*T_c_*), crystallization enthalpy (Δ*H_c_*), melting point (*T_m_*), melting enthalpy (*ΔH_m_*) and the calculated values of the crystallization degree (χ) were presented in Table 5.

Obtaining a homogeneous blend consisting of PLA and PEG depends to a large extent on the molecular mass of PEG. Information available in literature [46,47] indicates that blends of PLA with PEG1500 separate when the content of poly(ethylene glycol) exceeds 20 wt.%. For this reason it is reasonable to assume, that the obtained films present a homogeneous structure.

Based on the obtained results it can be seen that the increase in the amount of PEG causes a significant decrease in the glass transition temperature as well as crystallization temperature values. It was established by Piórkowska et al. [48] that the plasticizer molecules in the form of PEG significantly increase the free volume of the mixture, consequently allowing cooperative movements of macromolecules and, for this reason, decreasing the *T_g_* value. Simultaneously, plasticization of PLA by means of PEG intensifies the crystallization of PLA and in this way lowers its *T_c_*. Moreover, with the increase in PEG load, higher values of melting enthalpy and degree of crystallinity can be observed. The introduction of quercetin into the PLA-PEG systems results in an opposite effect. It has to be stressed that the presence of quercetin leads to a shift in glass transition temperature and crystallization temperature to higher values. Simultaneously, it can be observed that the values of both enthalpies, crystallization and melting, increase with the rise in the quercetin content. Furthermore, it should be noted, that quercetin has a profound impact on the degree of crystallinity. The direct correlation between the amount of quercetin and the degree of crystallinity of the obtained materials is apparent. This finding is consistent with the FTIR results, where the intensity of the band assigned to the crystalline phase decreases with the increase in quercetin content in the PLA-based films.

Moreover, the tests reveal that in the case of materials containing 10% wt of PEG, incorporation of quercetin causes the formation of different crystalline PLA forms. This phenomenon was widely described by Tabi et al. [49]. The formation of two crystalline forms is not observed in the case of polymeric films consisting of PLA, 5% PEG and quercetin, which is well worth noting.

### 3.4. Evaluation of Mechanical Properties

**Figure 11:**
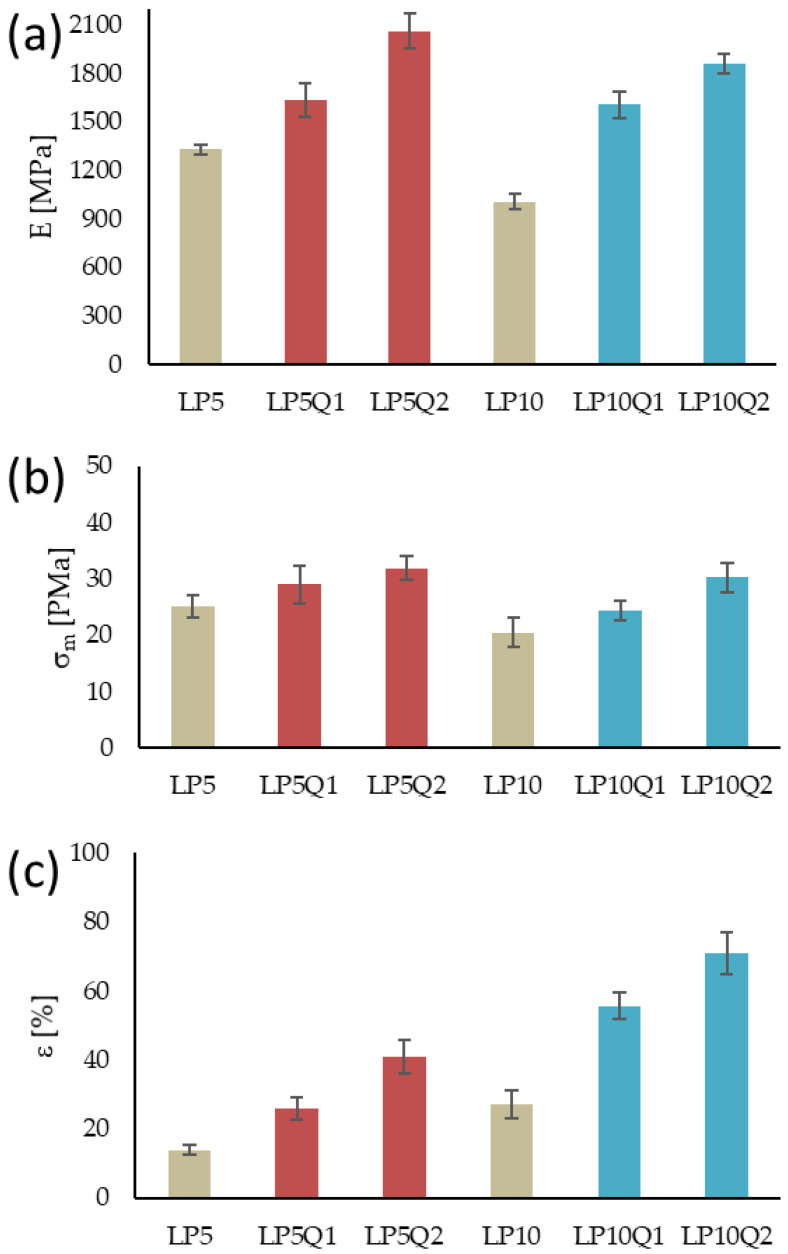
Mechanical properties of polylactide-based materials: (**a**) Young’s modulus (E), (**b**) Tensile strength (*σ_m_*), (**c**) Elongation at break (*ε*).

It is a commonly recognized fact that mechanical properties of packaging materials are extremely important [16]. In order to establish the impact different amounts of plasticizer and biocidal agent in form of quercetin have on the mechanical properties, the Young’s modulus (*E*), tensile strength (*σ_m_*) and elongation at break (*ε*) were determined. The values of Young’s modulus and tensile strength, are shown in Figure 11a,b, respectively. In the case of a sample with a higher PEG content (LP10) a significant decrease of the Young’s modulus and tensile strength in comparison with LP5 sample has been observed. These observations are consistent with results obtained by other researches [50,51] in relation to similar composition. During the study it has been established that the decrease in tensile strength, as well in Young’s modulus values, can be attributed to poor stress transfer between the PLA and PEG phases [52]. An introduction of quercetin into polylactide-PEG systems significantly improves the properties indicated above. The PLA-PEG samples containing 1 as well as 2 wt.% of quercetin was characterized by a remarkable reinforcement of their structure. The highest values of mechanical properties were obtained in relation to the LP5Q2 sample. The value of Young’s modulus for this material was about 2060 MPa while the maximum tensile strength reached about 32 MPa. The elongation at break (*ε*) values are presented in Figure 11c.

It can be clearly seen that an increase in the elasticity is correlated with the increase in the PEG content. It is well known that this phenomenon is related to reduction of intermolecular friction between the polymer molecules caused by the plasticizer molecules. The addition of quercetin allowed obtaining films with higher values of elasticity compared to the LP5 and LP10 samples. The highest value of the elongation at break was recorded for LP10 filled with 2.0 wt.% of quercetin. Moreover, it has to be stressed that in the case of the LP10Q2 sample the values of Young’s modulus and elongation at breaking point are twice as high as for the LP10 material. Taking into account the obtained results the plasticizing effect of quercetin could potentially seem surprising. The same tendency, however, was reported in literature [53,54]. In the work of Luzi et al. [55] it was established that an improvement of the mechanical properties can be related to the inter-molecular interactions between hydrophilic groups of the polymer matrix and the polyhydroxyl groups of quercetin. In the work of K. Rubini et al. [53], where the influence of quercetin on the mechanical properties of gelatin-based films was evaluated, the values of the Young’s modulus, the stress at break and the deformation at break increased significantly up to a point in which the flavonoid concentration of 1.5 wt.% was achieved. This limitation was caused by the non-homogeneous distribution of quercetin in gelatin. The susceptibility to deformation was also observed in the work of M. Latos-Brozio and A. Masek [54] where mechanical properties of PLA impregnated with quercetin and other substances of plant origin were discussed. Based on the obtained results and indicated literature, it is reasonable to assume that an incorporation of quercetin into the PLA matrix leads to an increase in mechanical properties of the obtained polymeric films.

### 3.5. Thickness and Transparency of Studied Materials

The values of thickness and transparency were established based on the absorbance measurements and presented in Table 6. The obtained results indicate that an increase in the amount of PEG significantly increases the thickness of LP10 film in comparison with the LP5 sample. These findings are consistent with the results presented in literature. Anuar et al. [56] have established that an introduction of PEG into the polylactide matrix caused a diffusion of the plasticizer between the PLA chains. As a result, an increase of free volume between polylactide chains as well as an increase of thickness can be observed. Incorporation of quercetin into the PLA-PEG systems slightly decreases the thickness of the obtained materials, which is quite unexpected. In most cases, the introduction of different types of substances, such as essential oils, into the polymer matrix results in an increase of material thickness [16,57,58]. In the case of quercetin the opposite effect has been observed. Presumably, this can be connected to the interactions between –COOH groups of PLA and -OH of quercetin.

Taking into account the results presented in Table 6, it was established that an addition of 10 wt.% of poly(ethylene glycol) significantly decreases the transparency in comparison with a polymeric film containing 5 wt.% of PEG. Observations indicated that the transparency of the LP5 films is twice as high as that of LP10. This is consistent with literature [58], where in the case of materials containing 20 wt.% of poly(ethylene glycol), the transparency value of the PLA-PEG system achieved a value of 2.63. Thus, it can be assumed the transparency of blends consisting of PLA and PEG significantly depends on the composition. An introduction of 1 wt.% as well as 2 wt.% of yellow quercetin significantly decreases transparency of the obtained materials. It should be stressed, however, that the decrease in transparency of films infused with the same amount of quercetin (LP5Q1, LP10Q1 as well as LP5Q2 and LP10Q2) has similar values regardless of the polymer compositions (LP5 and LP10). This phenomenon clearly indicates that in the case of studied materials transparency is mainly related to the amount of used quercetin rather than the applied PLA-PEG system. The obtained results suggest that polymeric films containing quercetin can be used as packaging materials, especially for products which are light sensitive. Summarizing, the application of materials consisting of PLA, PEG and quercetin will not allow for discoloration of products, loss of flavor or nutrients.

### 3.6. Differences in Colour of Studied Materials

In order to improve the product’s appearance, which significantly influences the consumer’s decision-making process in terms of purchase, the packaging materials are mixed with compounds which are able to change their color. In this paper the color of the polymeric films based on PLA with an addition of poly(ethylene glycol) as plasticizer and quercetin as antibacterial agent was analyzed. The values of measured parameters *L*, *a*, *b* and the calculated parameters such as total color difference (Δ*E*), chroma (*C*) and yellowness index (*YI*) are listed in Table 7.

In the case of samples consisting of PLA and PEG, the color difference equals 0.9 and proves that an addition of a higher amount of plasticizer does not influence the color changes. According to literature [33] the values of Δ*E* lower than 3 indicate that only an experienced observer is able to notice the difference in color of the studied materials. The changes in color, however, were observed in the case of films comprising quercetin. The changes in color of the obtained materials were not intentional and were most likely caused by an addition of a yellow—quercetin which significantly influenced the appearance of the samples. It can be clearly seen that with an increasing amount of quercetin the PLA-based films seem to be darker. Moreover, the color shifts to yellow upon increasing the amount of quercetin. The most significant color changes are observed after introducing 2 wt.% of an antibacterial agent into the LP5 and PL10 systems. In the case of the LP5Q2 sample Δ*E* equaled 31.9, while for the LP10Q2 film Δ*E* equaled 33.6. The same tendency was observed in the case of topas cyclo-olefin copolymer (ethylene-norbornene) with an addition of quercetin described in the work of A. Masek et al. [25].

The second color parameter analyzed in this work was chroma (*C*) which is strongly connected with colorfulness. It has to be stressed that the higher the chroma values, the higher is the color intensity of the analyzed materials indicated by observers [33]. It is not surprising, that in the case of obtained materials with an addition of quercetin the chroma value significantly increased. This phenomenon is connected with the yellow color which is assigned to the conjugation in the B-ring cinnamoyl system and is typical of flavonoids [59]. Similar observations can be made in the case of the yellowness index (*YI*) values. Significant differences in *b* and, as a result, in *C*, Δ*E* and *YI* values were established between samples with and without quercetin. Summarizing, the addition of quercetin into PLA-PEG systems significantly influences the color parameters of the obtained materials.

### 3.7. Examination of Antibacterial Properties

It is a widely recognized fact that improving the antibacterial properties of biodegradable packaging materials prevents the development of food-damaging pathogens and significantly reduces the volume of plastic waste, by providing extended shelf life. In order to ensure antibacterial properties of packaging materials, different compounds are introduced into the polymer matrix. Depending on the type of active additive, they can be divided into chemoactive and bioactive substances. The most chemoactive ingredients include iron, titanium and zinc. The characteristic feature of the compounds mentioned above is reactivity with oxygen. It should be stressed, however, that all of them can affect the chemical composition of the product as well as the interior environment of the package. In addition, they can cause adverse health effects and hinder the recycling process. The indisputable disadvantages of chemoactive compounds lead to a more extensive application of bioactive additives derived from natural sources, such as polyphenols, essential oils and other natural extracts [15,16,17]. These substances play an important role in active packaging. As antioxidants, they allow to reduce production costs and eliminate the risk related to food safety. In the present work, the disk-diffusion method was applied in order to establish the capacity to combat microorganisms characterizing the obtained materials imbued with quercetin. In Table 8 the results of antibacterial properties are presented. In the case of the LP5 and LP10 films assigned as control samples, the inhibition of *S. Aureus* and *E. coli* growth was not observed.

**Figure 12:**
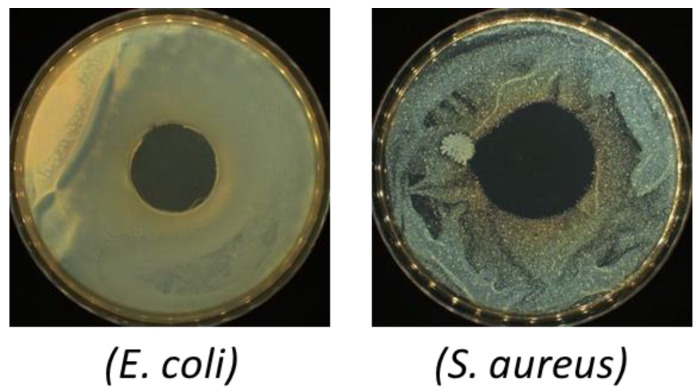
Photos of Bacteria (*E. coli* and *S. aureus*) growth in direct contact with LP10Q2 material.

The same tendency was observed in literature [58,60]. Introduction of quercetin into the PLA-PEG systems significantly changed the antibacterial properties of the obtained materials (Figures S1 and S2). Moreover, it was established that quercetin creates a more significant obstacle for the microorganisms in the case of materials containing a higher amount of plasticizer (LP10Q2) (Figure 12). It is, therefore, reasonable to assume that the mentioned above beneficial change resulted from an increase of free volume between PLA chains caused by PEG. The effect of poly(ethylene glycol) on the antibacterial properties was also evaluated in our previous publication [16].

In literature [61,62] quercetin is considered to be a biocidal agent. Ohemeng et al. [63] have established that quercetin is able to inhibit the *E. coli* DNA gyrase. Plaper et al. [64] showed that quercetin forms a bond with the GyrB subunit of *E. coli* DNA gyrase. Moreover, additional antibacterial mechanisms, triggered by quercetin, resulted in destabilizing of bacterial membrane functions were described by Mirzoeva et al. [65]. It should be stressed that the reported antibacterial activity of materials containing quercetin is consistent with the data published by Kost et al. [26]. Recent studies as well as the obtained results clearly indicate that quercetin has the potential to combat bacteria and for this reason the incorporation of quercetin contributed to an increase in antibacterial capacity of the obtained materials.

## 4. Conclusions

In summary, the influence of quercetin on the physico-chemical and antibacterial properties of PLA based materials was examined. Quercetin was introduced into two polymer systems consisting of polylactide and poly(ethylene glycol) (5 and 10 wt.%, respectively). The obtained materials containing quercetin were characterized by a significant improvement in thermal and mechanical properties. Simultaneously, it was also proved that the observed changes are a result of the inter-molecular interactions between hydrophilic groups of polylactide and poly(ethylene glycol) with the polyhydroxyl groups of quercetin. Moreover, it should be stressed that the introduction of quercetin allows obtaining antibacterial polymeric films. Furthermore, it can be clearly noticed that the yellow color of quercetin influences the change in the color and transparency of the studied materials. The films consisting of PLA, PEG and quercetin constitute promising bactericidal materials which can be applied as packaging materials.

## Figures and Tables

**Table 1:** Compositions of the obtained materials (L-polylactide; P-PEG, Q-quercetin).

Sample	Quercetin Content ^1^ [wt.%]	PEG Content ^1^ [wt.%]
LP5	-	5
LP10	-	10
LP5Q1	1	5
LP10Q1	1	10
LP5Q2	2	5
LP10Q2	2	10

^1^ Relative to PLA mass.

**Table 2:** Antibacterial effect of antibacterial treatment (ISO 20645, 2006) [34].

Braking Zone [mm] the Average Value of Rise	Growth ^(a)^	Description	Rating
>1	Lack	Inhibition zone above 1, no increase ^(b)^	Good effect
0–1	Lack	Growth inhibition zone up to 1, no growth ^(b)^	
0	Lack	No braking zone, no increase ^(c)^	
0	Weak	Lack of braking zones, only some colonies limited growth almost completely stopped ^(d)^	Limited effect
0	Average	No braking zone, height reduced to half compared to control ^(e)^	
0	Strong	Lack of braking zones, the absence of a reduction in growth compared to the control, or only a slight reduction in growth	Insufficient effect

^(a)^ Bacterial growth on the medium under the working sample. ^(b)^ The dynamometer range should only be partially taken into account in the calculations. An increase in the braking zone may be due to excess of active substance or unevenness of the substance in the article. ^(c)^ Lack of growth with a simultaneous lack of braking zone can be considered a good effect. A braking zone may not be possible due to limited diffusion. ^(d)^ “Almost as good as lack of growth”—an indication of limited efficiency. ^(e)^ Limited bacterial growth density means both the number of colonies and the diameter of the colonies.

**Table 3:** Roughness parameters of PLA and PLA-based films with and addition of quercetin and poly(ethylene glycol).

Sample	*R_q_* [nm]	*R_a_* [nm]	*R_max_* [nm]
LP5	1.4 ± 0.1	1.0 ± 0.1	10.5 ± 0.5
LP10	3.8 ± 0.2	3.1 ± 0.2	23.1 ± 1.1
LP5Q1	32.2 ± 0.5	22.0 ± 0.4	232.0 ± 2.0
LP5Q2	34.1 ± 0.6	26.8 ± 0.5	237.0 ± 2.0
LP10Q1	69.5 ± 1.2	51.4 ± 1.0	344.0 ± 4.0
LP10Q2	71.9 ± 1.4	55.40 ± 1.0	364.0 ± 7.0

**Table 4:** TG data for obtained materials with and without addition of quercetin.

Samples	Temperature (°C) at Mass Loss		
	10%	30%	50%
LP5	289.5 ± 0.4	340.1 ± 1.5	353.7 ± 1.5
LP10	287.2 ± 0.5	335.6 ± 1.3	350.9 ± 1.4
LP5Q1	322.6 ± 0.7	347.9 ± 1.0	358.5 ± 1.4
LP5Q2	329.8 ± 1.2	348.3 ± 1.0	358.3 ± 1.6
LP10Q1	320.0 ± 1.2	340.8 ± 1.3	352.4 ± 1.7
LP10Q2	325.5 ± 1.1	345.1 ± 1.3	356.1 ± 1.7

**Table 5:** DSC parameters for the all studied PLA-based materials.

Sample	*T_g_* [°C]	*T_c_* [°C]	Δ*H_c_* [J/g]	*T_m_* [°C]	Δ*H_m_* [J/g]	χ_m_ [%]
LP5	54.6 ± 0.2	101.4 ± 0.5	18.4 ± 0.3	146.8/153.4 ± 0.2/0.4	24.1 ± 0.4	23.3
LP5Q1	54.5 ± 0.2	112.4 ± 0.7	16.1 ± 0.1	153.8 ± 0.2	22.0 ± 0.4	21.5
LP5Q2	58.6 ± 0.4	119.6 ± 0.8	15.4 ± 0.1	152.3 ± 0.2	21.0 ± 0.3	19.7
LP10	49.5 ± 0.1	95.5 ± 0.5	17.9 ± 0.2	153.5 ± 0.3	26.6 ± 0.5	27.1
LP10Q1	54.0 ± 0.2	106.5 ± 0.6	15.0 ± 0.2	146.2/152.6 ± 0.2/0.3	24.8 ± 0.4	25.6
LP10Q2	54.8 ± 0.2	108.9 ± 0.6	12.8 ± 0.1	146.7/152.8 ± 0.3/0.3	16.6 ± 0.2	17.3

**Table 6:** Thickness and transparency values of studied materials.

Sample	Thickness [mm]	T [mm^−1^]
LP5	0.071 ± 0.002	0.76 ± 0.1
LP10	0.086 ± 0.002	1.54 ± 0.1
LP5Q1	0.065 ± 0.003	3.34 ± 0.1
LP5Q2	0.066 ± 0.001	6.04 ± 0.1
LP10Q1	0.070 ± 0.004	3.92 ± 0.1
LP10Q2	0.080 ± 0.004	6.58 ± 0.2

**Table 7:** Color (*L*, *a*, *b*, Δ*E*, *C*, *YI*) parameters of obtained PLA-based materials.

Sample	*L*	*a*	*b*	Δ*E*	*C*	*YI*
LP5	91.7 ± 0.1	1.5 ± 0.1	−11.6 ± 0.2	−	11.7 ± 0.2	−18.1 ± 0.2
LP10	91.5 ± 0.1	1.5 ± 0.1	−12.5 ± 0.3	0.9 ± 0.2	12.6 ± 0.2	−19.5 ± 0.4
LP5Q1	89.5 ± 0.2	−5.0 ± 0.1	13.4 ± 0.2	25.9 ± 0.4	14.3 ± 0.3	21.4 ± 0.5
LP5Q2	88.2 ± 0.3	−8.9± 0.2	18.4 ± 0.5	31.9 ± 0.4	20.4 ± 0.7	29.8 ± 0.8
LP10Q1	89.2 ± 0.1	−6.6 ± 0.4	14.9 ± 0.3	27.8 ± 0.3	16.3 ± 0.7	23.9 ± 0.3
LP10Q2	87.9 ± 0.2	−8.6 ± 0.3	20.2 ± 0.4	33.6 ± 0.2	22.0 ± 0.5	32.8 ± 0.8

**Table 8:** Results of antibacterial activity of the obtained PLA-based materials.

Sample	Diameter of Inhibition Zones of Bacteria Growth [mm]		Bacteria Growth in Direct Contact with Sample		Evaluation of Antibacterial Effect ^1^	
	*S. aureus*	*E. coli*	*S. aureus*	*E. coli*	*S. aureus*	*E. coli*
LP5	0	0	Medium	Medium	Insufficient	Insufficient
LP10	0	0	Medium	Medium	Insufficient	Insufficient
LP5Q1	0	0	Lack	Weak	Good	Good
LP5Q2	1	0	Lack	Lack	Good	Good
LP10Q1	0	0	Lack	Weak	Good	Limited
LP10Q2	4	1	Lack	Lack	Good	Good

^1^ in accordance with ISO 20645:2006 standard.

## Data Availability

The data presented in this study are available on request from the corresponding author.

## Supplementary Materials

The following are available online at https://www.mdpi.com/article/10.3390/ma14071643/s1, Figure S1: Photos of Bacteria (*E. coli* and *S. aureus*) growth in direct contact with samples containing 5% wt. of PEG, Figure S2: Photos of Bacteria (*E. coli* and *S. aureus*) growth in direct contact with samples containing 10% wt. of PEG.
