# Supplementary material for: Antibacterial Films Based on Polylactide with the Addition of Quercetin and Poly(Ethylene Glycol)
Source: Materials (Basel). 2021 Mar 27;14(7):1643. doi: 10.3390/ma14071643 (PMC8036468; doi:10.3390/ma14071643)
Supplement: Supplementary file 1 [file materials-14-01643-s001.pdf]

materials

MDPI

Supplementary Material

Antibacterial Films Based on Polylactide with the Addition of Quercetin and Poly(Ethylene Glycol)

Ewa Olewnik-Kruszkowska [1,*], Magdalena Gierszewska [1], Agnieszka Richert [2], Sylwia Grabska-Zielińska [1], Anna Rudawska [3] and Mohamed Bouaziz [4]

[1] Faculty of Chemistry, Chair of Physical Chemistry and Physicochemistry of Polymers, Nicolaus Copernicus University in Toruń, Gagarin 7 Street, 87-100 Toruń, Poland; mgd@umk.pl (M.G.); sylwia.gz@umk.pl (S.G.-Z.)
[2] Faculty of Biological and Veterinary Sciences, Chair of Genetics, Nicolaus Copernicus University in Toruń, Lwowska 1 Street, 87-100 Toruń, Poland; a.richert@umk.pl
[3] Faculty of Mechanical Engineering, Department of Production Engineering, Lublin University of Technology, 20-618 Lublin, Poland; a.rudawska@pollub.pl
[4] Electrochemistry and Environmental Laboratory, National Engineering School of Sfax, University of Sfax, BP1173, Sfax 3038, Tunisia; mohamed.bouaziz@fsg.rnu.tn
* Correspondence: olewnik@umk.pl; Tel.: +48-56-611-2210

**(a)** *(E. coli)*	**(b)** *(S. aureus)*
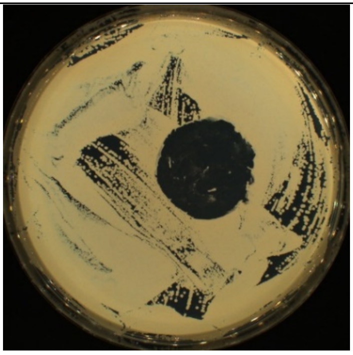 LP5	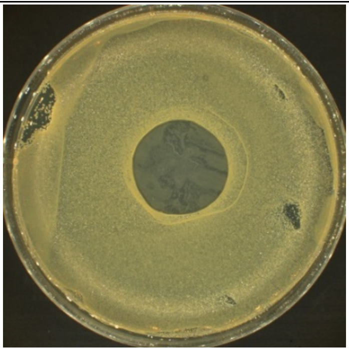 LP5
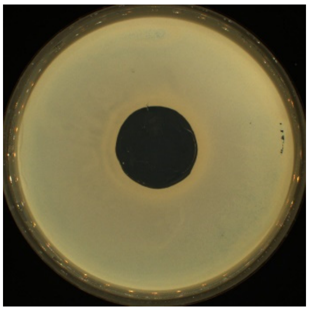 LP5Q1	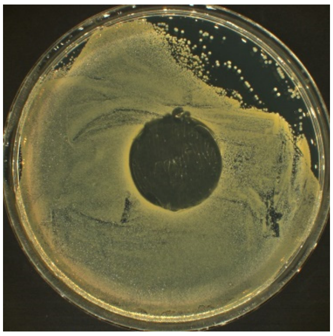 LP5Q1

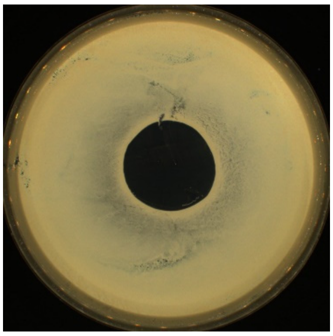	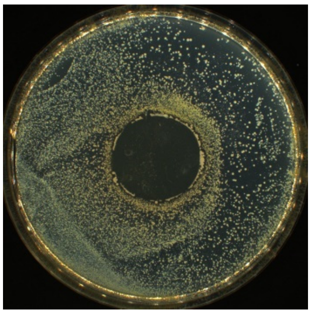
LP5Q2	LP5Q2

Figure S1. Photos of Bacteria (**a**) E. coli and (**b**) S. aureus growth in direct contact with samples containing 5% wt. of PEG.

**(a)** ***(E. coli)***	**(b)** ***(S. aureus)***
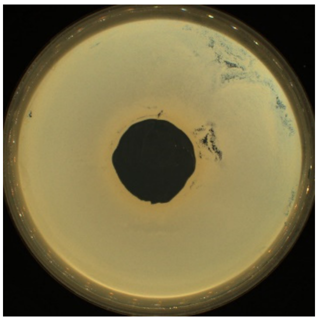	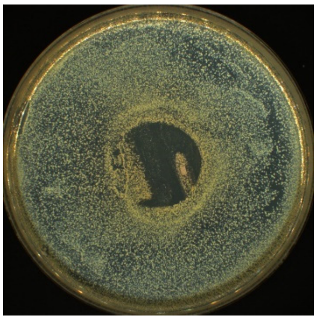
LP10	LP10
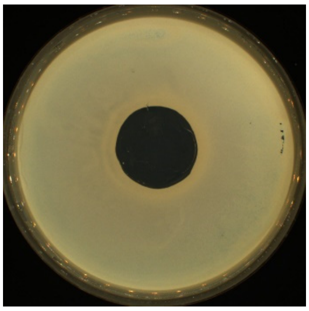	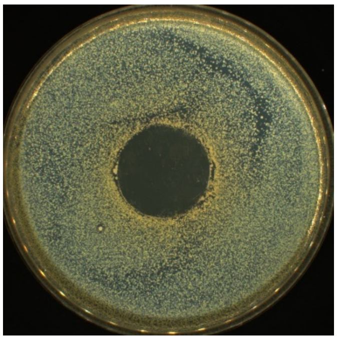
LP10Q1	LP10Q1
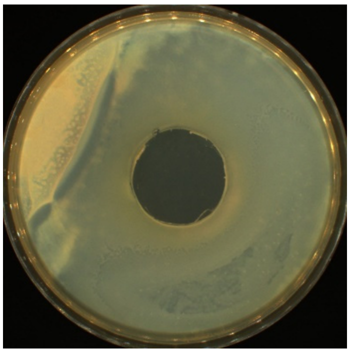	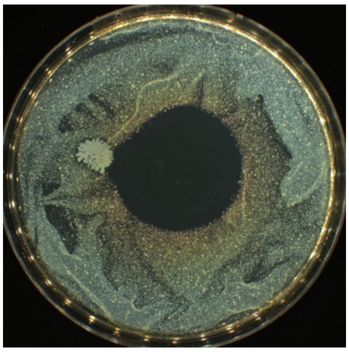
LP10Q2	LP10Q2

Figure 2. Photos of Bacteria (**a**) *E. coli* and (**b**) *S. aureus* growth in direct contact with samples containing 10 wt.% of PEG**.**
